# Lizard predation by spiders: A review from the Neotropical and Andean regions

**DOI:** 10.1002/ece3.6801

**Published:** 2020-09-22

**Authors:** Claudio Reyes‐Olivares, Andrés Guajardo‐Santibáñez, Bernardo Segura, Nicolás Zañartu, Mario Penna, Antonieta Labra

**Affiliations:** ^1^ Programa de Doctorado en Ciencias, con mención en Ecología y Biología Evolutiva Facultad de Ciencias Universidad de Chile Santiago Chile; ^2^ Laboratorio de Neuroetología Instituto de Ciencias Biomédicas Facultad de Medicina Universidad de Chile Santiago Chile; ^3^ Valparaíso Chile; ^4^ Flora y Fauna Chile Ltda. Santiago Chile; ^5^ Programa de Agronomía Facultad de Agronomía e Ingeniería Forestal Pontificia Universidad Católica de Chile Santiago Chile; ^6^ Centre for Ecological and Evolutionary Synthesis (CEES) Department of Biosciences University of Oslo Oslo Norway

**Keywords:** *Anolis*, Central Chile, Ctenidae, *Liolaemus*, predator–prey interactions, Theraphosidae

## Abstract

Vertebrate predation by invertebrates has been classically underexplored and thus underestimated, despite the fact that many arthropods consume vertebrates. To shed some light on the relevance that spider predation may have upon lizards in the Neotropical and Andean regions, we compiled the available information in the literature on this trophic interaction. We found 50 reports of spiders consuming lizards in these regions, and the 88% of these were from the Neotropical region. Spiders belong to eight families, but Ctenidae and Theraphosidae were the most frequently reported predators. Lizards belong to 12 families, and the most commonly consumed species corresponded to the families Dactyloidae (all *Anolis* lizards), Gymnophthalmidae, and Sphaerodactylidae. Data suggest trophic spider–lizard associations between Ctenidae and Dactyloidae, followed by Theraphosidae and Liolaemidae. The body sizes of the spiders and lizards showed a positive relationship, and spiders were smaller than their prey. We conclude that various spider taxa can be considered lizard predators and they may be ecologically important in the Neotropical and Andean regions. However, spiders of prime predation relevance seem to be those of the Ctenidae and Theraphosidae families.

## Graphical abstract

In this study, we analyze for the first time the lizard consumption by spiders in the Neotropical and Andean regions, showing that the families Ctenidae and Theraphosidae may be relevant lizard predators in these areas. In addition, we provide the first report on spider predation upon a vertebrate taxon in the Andean region.

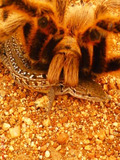

## 1 INTRODUCTION

In terrestrial environments, the study of predator–prey interactions has traditionally focused on vertebrate consuming invertebrates (Elewa, 2007; Jędrzejewska & Jędrzejewski, 2013; Taylor, 1984). In many cases, however, intraguild predation occurs (e.g., Rubbo et al., 2001) and thus, this predation interaction is reversed, as many arthropods such as arachnids, chilopods, insects, and crustaceans, prey upon small or medium‐sized vertebrates (reviewed by McCormick & Polis, 1982; Valdez, 2020). In spite of this accrue of information on arthropods consuming vertebrates, the incidence of predator–prey interactions of this kind remains relatively unexplored and thus underestimated (Nordberg et al., 2018).

Among arthropods, spiders are considered one of the main vertebrate predators (Valdez, 2020). In fact, spiders consume different taxa including fish (Nyffeler & Pusey, 2014), anurans (Nyffeler & Altig, 2020), lizards (Bauer, 1990; O’Shea & Kelly, 2017), snakes (Jorge et al., 2016), birds (Brooks, 2012), and mammals (Nyffeler & Knörnschild, 2013). Different characteristics allow spiders to consume such diversity of vertebrates, including the possession of strong fangs that can pierce vertebrate skin and inoculate paralyzing neurotoxins (Foelix, 2011; Garb & Hayashi, 2013). In addition, spiders have a generalist diet (Riechert & Harp, 1987) and a variety of hunting modalities, ranging from active foraging to ambush or sit‐and‐wait strategies (Willemart & Lacava, 2017), combined with the ability to build webs to trap prey (Foelix, 2011; Gosline et al., 1999). All these characteristics, in addition to the fact that spiders can have larger body size than some vertebrate species, allow them to prey upon different vertebrate taxa (McCormick & Polis, 1982; Vieira et al., 2012).

The information on predator–prey interactions between spiders and lizards is scattered, and although some attempts have been made to systematize this information (Bauer, 1990; O’Shea & Kelly, 2017; Schalk & Cove, 2018; Valdez, 2020), we have a very limited understanding whether lizard consumption by spiders is relevant in shape lizard features, for example, the evolution of some defenses (Schalk & Cove, 2018). This contrast with the available knowledge on spiders consuming other vertebrate taxa, which provide insights of the occurrence and extension of these interactions across the world, that is, fish (Nyffeler & Pusey, 2014), amphibians (Menin et al., 2005; Nyffeler & Altig, 2020), and bats (Nyffeler & Knörnschild, 2013). The scarcity of organized information for lizards is particularly striking when considering regions having a high diversity of lizard and spider species, such as the Neotropics (Pianka & Vitt, 2003; Santos et al., 2017). Although instances of consumption of lizards by spiders have been reported for this region (e.g.,Clark & Gillingham, 1990; Folt & Lapinski, 2017; Gomides et al., 2010; von May et al., 2019), the information has not been systematized, which hinders a comprehensive assessment of the significance of spider–lizard predator–prey interactions. In the current study, we compiled all the available information in the literature on predation events involving these two taxa, to organize the occurrence of spider consuming lizard reports in the Neotropical region. This allows evaluating the extension of these predator–prey interactions in a geographical area characterized by its high biological diversity. This information combined with a determination of which are the taxa involved in these interactions is a critical first step to understand how spiders can modulate lizard populations, and so, their communities.

Originally, the Neotropical region was defined according to the Sclater–Wallace system, to comprise South America and part of Central America, extending northbound as far as central Mexico (Sclater, 1858; Wallace, 1876). This scheme has been widely accepted by different zoogeographers studying vertebrate distribution (Cox, 2001; Morrone, 2014, 2015; Rueda et al., 2013). Other authors, however, especially those interested in invertebrate biogeography, have excluded from the Neotropical region the Andean area and the southern part of South America, as these regions are historically associated with other Austral areas, namely Australia, New Guinea, New Zealand, Tasmania, and South Africa (reviewed by Morrone, 2014, 2015). Because the current review focuses on invertebrate predators, we have considered the Andean region apart from the Neotropical region, as defined by Morrone (2014, 2015), compiling separately the records on lizard predation by spiders in these two regions.

## 2 MATERIALS AND METHODS

We conducted an exhaustive literature review on spiders consuming lizards in the Neotropical and Andean regions. We searched for information using Google Search, Google Scholar, Thomson‐Reuters, and Scopus databases. The keywords searched were spider, Arachnida, lizard, Lacertilia, predation, Neotropical, Andean, and Andes, used in different combinations. The quest was repeated using the same keywords in Spanish and Portuguese. Finally, we searched information throughout the entire collection of Herpetological Review (1967–June 2020). We considered a “predation event” reports on spiders hunting, seizing, and/or consuming a lizard. In addition, we included reports in which a dead lizard, trapped in a spider web or found in close proximity to a spider burrow, showed clear signs of been consumed by a spider, that is, mastication, envenomation, and/or external digestion.

We explored relationships between the sizes, activity patterns (diurnal vs. nocturnal), and hunting strategies (active forager vs. ambush predator) of predators and prey, obtaining these data from additional publications for all the species reported in our literature search. We searched for the adult sizes, snout–vent length for lizards, and carapace length for spiders, and this information was used for the analyses, because these data were not always available in the reports. For species that exhibited sexual dimorphism, we reported the body sizes of both sexes when possible, although we restricted the analyses to the females’ body sizes. The information on activity patterns and hunting strategies may help to assess the probabilities that predator and prey come in contact with each other (Miller et al., 2014).

We constructed a heat map based on the number of lizard species by family that was consumed by spiders of different families. The relationship between the predator–prey body sizes (log transformed) was evaluated with a Pearson correlation, and the body size differences between predator and prey were assessed with a *t* test. The analyses that included the body sizes were restricted to cases in which predator and prey species were identified. A Fisher's exact test was performed to assess relationships of the activity patterns and foraging strategies between predators and prey, excluding those events that happened in a trap, as this was an artificial situation.

## 3 RESULTS

Table 1 summarizes 50 reports spiders consuming lizards in the Neotropical and Andean regions (Table 1); 47 come from the literature, and three are new records communicated in this study (Table 1). Most reports are based on a single observation (91%), and the rest included two observations. None of these reports provide experimental data on the impact that spider predation may have upon lizards or on the frequency of occurrence of these events. The predator attack was observed in only five cases (10%; Table 1), while the rest of the reports only describe from the prey consumption. In four of these 45 cases (8.9%), lizards were found trapped in spider webs of the Araneidae family, and in six cases (13%), the spider was found consuming the prey inside a trap (Table 1).

**TABLE 1:** Reports of the predatory events by spiders upon lizards in the Neotropical and Andean regions

Lizard (family/species)	SVL (mm)/sex/age	Spider family	Spider species	W (g)/CL (mm)/TL (mm)	Attack observed	Country	Source
**Alopoglossidae**							
*Ptychoglossus bicolor*	45/‐/‐	**Theraphosidae**	*Pamphobeteus ferox*	‐/‐/55‐65	No	Colombia	[1]
**Dactyloidae**							
*Anolis sp*.	‐/‐/J	**Salticidae**	*Phidippus cf. bidentatus*	–	No	Costa Rica	[2]
*Anolis chrysolepis*	30/‐/J	**Ctenidae**	*Ctenus sp*.	–	No	French Guiana	[3]
*Anolis fuscoauratus (= Norops fuscoauratus)*	28/‐/J	**Ctenidae**	*Ctenus sp*.	–	No	Colombia	[4]
*Anolis gundlachi*	17/‐/‐	**Ctenidae**	*Ctenus ottleyi*	2.28/‐/‐	No	Puerto Rico	[5]
*Anolis humilis*	–	**Ctenidae**	–	–	–	Costa Rica	[6]
*Anolis humilis*	16/‐/J	**Ctenidae**	*Kiekie curvipes* (=*Ctenus curvipes*)	0.15/‐/‐	Yes	Costa Rica	[7]
*Anolis humilis*	‐/F/J	**Trechaleidae**	*Cupiennius sp*.	14/‐/‐	No	Costa Rica	[7]
*Anolis limifrons*	–	**Trechaleidae**	*Cupiennius sp*.	–	No	Panama	[8]
*Anolis limifrons*	–	**Salticidae**	–	–	No	Costa Rica	[9]
*Anolis porcatus*	<30/‐/J	**Araneidae**	*Argiope trifasciata*	–	No^a^	Cuba	[10, cited by 11]
*Anolis rodriguezii*	‐/M/A	**Sparassidae**	–	–	No	Mexico	[12]
*Anolis sagrei*	<30/‐/J	**Araneidae**	*Argiope trifasciata*	–	No^a^	Cuba	[10, cited by 11]
*Anolis sagrei*	31/M/J	**Trechaleidae**	*Cupiennius cf. cubae*	0.94/8.6/‐	No	Cuba	[13]
**Gekkonidae**							
*Hemidactylus mabouia*	45.5/‐/‐	**Araneidae**	*Nephilengys cruentata*	‐/‐/25.6	No^a^	Brazil	[14]
*Hemidactylus mabouia*	133.9/‐/‐	**Ctenidae**	Unidentified	‐/‐/20	No	Brazil	[15]
*Hemidactylus mabouia*	50.5/‐/J	**Lycosidae**	Unidentified	‐/‐/29	No	Brazil	[16]
*Hemidactylus mabouia*	50/‐/A	**Theraphosidae**	*Avicularia variegata*	‐/‐/120	No	Brazil	[17]
*Hemidactylus frenatus*	‐/F/‐	**Ctenidae**	*Phoneutria boliviensis*	–	No	Colombia	[18]
**Gymnophthalmidae**							
*Arthrosaura reticulata*	37.8/M/J	**Ctenidae**	*Ancylometes rufus*	‐/‐/32.9	Yes	Brazil	[19]
*Cercosaura eigenmanni*	–	**Ctenidae**	*Ctenus sp*.	–	Yes	Peru	[20]
*Cercosaura schreibersii*	‐/‐/A	**Theraphosidae**	*Plesiopelma sp*.	–	No	Uruguay	[21]
*Cercosaura schreibersii*	–	**Lycosidae**	*Lycosa erythrognatha*	–	No	Brazil	[22]
*Ecpleopus gaudichaudii*	24/‐/‐	**Ctenidae**	*Ctenus cf. ornatus*	–	No	Brazil	[23]
*Loxopholis guianense (=Leposoma guianense)*	‐/‐/A	**Ctenidae**	*Ctenus sp*.	–	No	French Guiana	[24]
*Micrablepharus atticolus*	–	**Lycosidae**	*Lycosa erythrognatha*	–	No	Brazil	[25]
*Micrablepharus maximiliani*	–	**Theraphosidae**	Unidentified	‐/‐/~6	No	Brazil	[26]
**Leiosauridae**							
*Enyalius bilineatus*	31.3/‐/J	**Ctenidae**	*Ctenus ornatus*	‐/28.2/‐	No^b^	Brazil	[27]
*Enyalius perditus*	31.8/‐/J	**Ctenidae**	*Ctenus ornatus*	‐/21.8/‐	No^b^	Brazil	[27]
**Liolaemidae**							
*Liolaemus lemniscatus*	‐/‐/A	**Theraphosidae**	*Grammostola rosea*	–	Yes	Chile	This study
*Liolaemus nigroviridis*	‐/‐/J	**Theraphosidae**	Unidentified	–	No	Chile	[28]
*Liolaemus nitidus*	‐/‐/J	**Theraphosidae**	*Euathlus sp*.	–	No	Chile	This study
*Liolaemus tenuis*	‐	**Theraphosidae**	*Grammostola rosea*	–	No	Chile	This study
**Phyllodactylidae**							
*Phyllodactylus gerrhopygus*	28/‐/J	**Sicariidae**	*Sicarius thomisoides*	‐/‐/20	No	Chile	[29]
**Scincidae**							
*Plestiodon sumichrasti*	60/‐/J	**Theraphosidae**	*Brachypelma sp*.	–	No	Guatemala	[30]
*Scincella cherriei*	23.7/‐/J	**Ctenidae**	*Anahita sp*.	‐/‐/17	No	Mexico	[31]
**Sphaerodactylidae**							
*Chatogekko amazonicus*	14.73/‐/J	**Ctenidae**	Unidentified	‐/‐/10.61	No	Brazil	[32]
*Coleodactylus meridionalis*	–	**Ctenidae**	*Parabatinga brevipes*	–	No	Brazil	[33]
*Coleodactylus meridionalis*	‐/‐/A	**Ctenidae**	*Ctenus rectipes*	–	No	Brazil	[34]
*Gonatodes albogularis*	‐/F/A	**Araneidae**	*Nephila clavipes*	–	No^a^	Costa Rica	[35]
*Gonatodes albogularis*	**‐**/F/**‐**	**Ctenidae**	*Phoneutria boliviensis*	–	No	Colombia	[18]
*Lepidoblepharis xanthostigma*	24.1/M/A	**Theraphosidae**	*Trichopelma sp*.	‐/29/‐	No	Colombia	[36]
**Teiidae**							
*Aurivela longicauda*	47.1/M/A	**Lycosidae**	*Lycosa poliostoma*	–	No^b^	Argentina	[37]
*Aurivela longicauda*	–	**Theraphosidae**	*Grammostola mendozae*	–	No^b^	Argentina	[38]
*Ameivula nigrigula*	‐/‐/J	**Theraphosidae**	*Lasiodora sp*.	–	No	Brazil	[39]
*Kentropyx striata*	–	**Theraphosidae**	*Theraphosa cf. blondi*	–	Yes	Brazil	[40]
**Tropiduridae**							
*Tropidurus hispidus*	‐/F/A	**Theraphosidae**	*Lasiodora klugi*	–	No	Brazil	[41]
*Tropidurus oreadicus*	783/‐/A	**Lycosidae**	*Lycosa erytrognata*	–	No^b^	Brazil	[42]
*Tropidurus semitaeniatus*	41.72/‐/J	**Theraphosidae**	*Acanthoscurria natalensis*	‐/‐/55	No^b^	Brazil	[43]
**Xantusiidae**							
*Lepidophyma tuxtlae*	28/‐/N	**Lycosidae**	*Hogna* sp.	‐/8/17	No	Mexico	[44]

Note

Families and species of predators and prey are listed. Size, weight, sex, and age of individuals involved in the predatory event are indicated when available. Also is listed the information of the attacks (yes/no), the country where the event was reported, and the corresponding reference.

1. Castillo‐Rodríguez and Méndez‐Galeano (2017); 2. Nyffeler et al. (2017); 3. de Massary and Ineich (1997); 4. Medina‐Rangel (2013); 5. Clark and Gillingham (1990); 6. Guyer (1988); 7. Folt and Lapinski (2017); 8. Bock and Quintero (1987); 9. Losos (2011); 10. Armas and Alayón (1987); 11. Armas (2001); 12. García‐Balderas et al. (2016); 13. Fonseca and Rodríguez‐Cabrera (2014); 14. Diniz (2011); 15. Lanschi and Ferreira (2012); 16. Koski et al. (2013); 17. Almeida et al. (2019); 18. Valenzuela‐Rojas et al. (2020); 19. Waldez and Lima (2006); 20. von May et al. (2019); 21. Borges et al. (2016); 22. Bressan et al. (2017); 23. Pereira‐Ribeiro et al. (2017); 24. de Massary (1999); 25. Maffei et al. (2010); 26. de Sousa and Freire (2010); 27. Gomides et al. (2010); 28. Troncoso (2010); 29. Taucare‐Ríos and Piel (2020); 30. Streicher et al. (2011); 31. Aguilar‐López et al. (2014); 32. Hernández‐Ruz et al. (2014); 33. Almeida et al. (2015); 34. Oliveira et al. (2017); 35. Filipiak and Lewis (2012); 36. Quintero‐Ángel and Carr (2010); 37. Galdeano et al. (2017); 38. Kass et al. (2018); 39. Leite et al. (2019); 40. Coêlho et al. (2019); 41. Vieira et al. (2012); 42. Bocchiglieri and Mendonça (2010); 43. Ferreira et al. (2014); 44. Cabrera‐Guzmán and Reynoso (2007).

Abbreviations: A, adult; F, female/age; J, juvenile; M, male; N, neonate; SVL/sex/age, snout–vent length in mm/sex; W (g)/CL (mm)/TL (mm): Weight (g)/Carapace length (mm)/Total body length (mm).

^a^ Prey captured in a spider web.

^b^ Predatory event occurred inside a trap (e.g., funnel, Sherman, pitfall).

**FIGURE 1:**
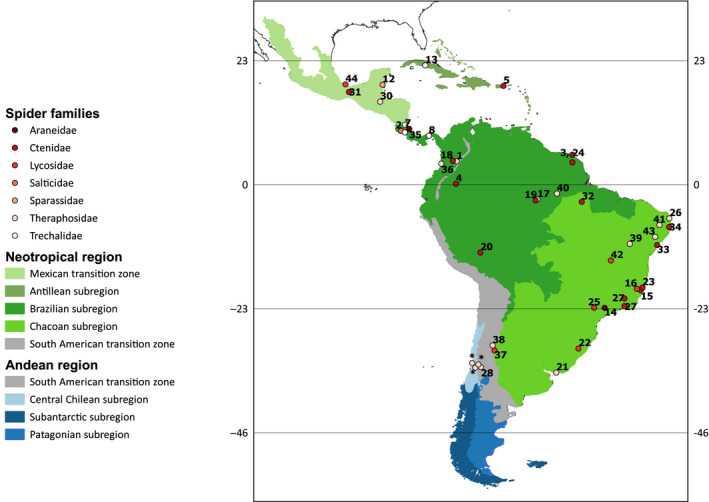
Geographical distribution of predation reports by spiders upon lizards (circles) in the Neotropical and Andean regions, showing the corresponding subregions (sensu Morrone, 2014, 2015). The spider families are indicated by circles of different colors, and numbers correspond to the references listed in Table 1. Asterisks correspond to the three new records reported in this study. Six reported records are not shown because their georeferenced location is missing (references 6, 9, and 10, cited by 11, 18, and 29, respectively; Table 1)

The geographical distribution of the predation reports is shown in Figure 1. Most cases (84%) were from the tropics (between 23°N to 23°S), while the rest were from the temperate zone of South America (>23° S; Figure 1). Most of the predation events (88%) were exclusively from the Neotropical region, while the rest were from the Andean Region (6%) and the South American transition zone (6%), the area between the Neotropical and the Andean regions (Table 1, Figure 1). Reports from the Neotropical region were distributed unevenly, as most of these were from two subregions, Chacoan (41%), along the Atlantic coast (i.e., Paraná Dominion, Morrone, 2014), and Brazilian (41%). These were followed by the Antillean region (9%) and the Mexican transition zone (9%) (Figure 1). In the Andean region, the few available reports were from the Central Chile subregion (Figure 1). Finally, the 50 observations included 13 countries (Table 1), among which Brazil had the highest proportion of reports (38%).

**FIGURE 2:**
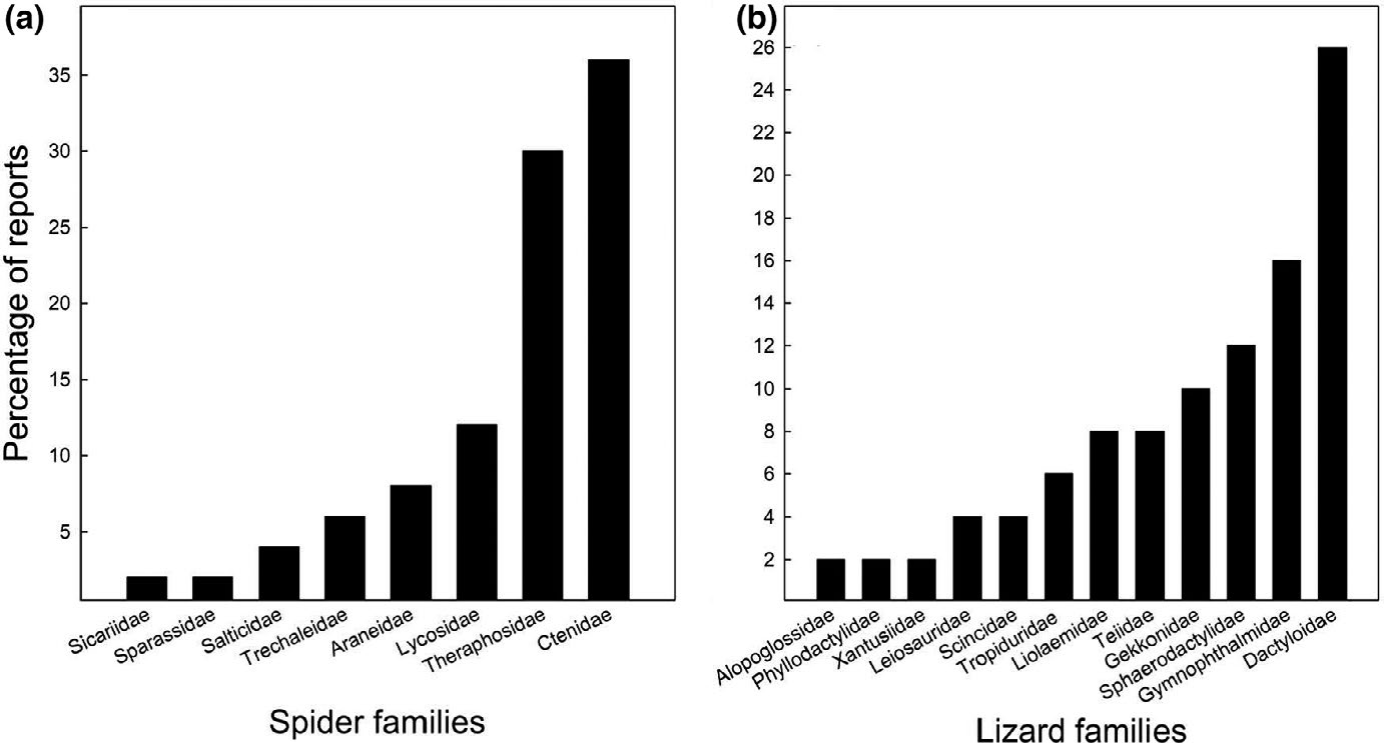
Percent of families of (a) spider predators and (b) lizard prey involved in the predator reports from the Neotropical and Andean regions

The predator spiders of lizards belong mostly to the suborder Araneomorphae, which includes seven families: Araneidae (orb‐weaver spiders), Ctenidae (wandering spiders), Lycosidae (wolf spiders), Salticidae (jumping spiders), Sicariidae (six‐eyed sicariid spiders), Sparassidae (huntsman spiders), and Trechaleidae (trechaleid spiders). The second suborder represented was Mygalomorphae, for which predation events only included the family Theraphosidae (tarantulas). The two main predator families were Ctenidae and Theraphosidae, and the less represented were the families Sparassidae and Sicariidae (Figure 2a).

Lizards consumed by spiders belonged to 12 families (Table 1, Figure 2b), from which the three main corresponded to Dactyloidae, Gymnophthalmidae, and Sphaerodactylidae (Figure 2b). From the 50 reports listed in Table 1, 32 (64%) included the lizard ages, and from these, 62.5% corresponded to juveniles, 34.4% to adults, and 3.1% to neonates (a single case). From the 38 lizard species listed in Table 1, eight had more than one predation event (two to four), and these involved different spider species (Table 1).

**FIGURE 3:**
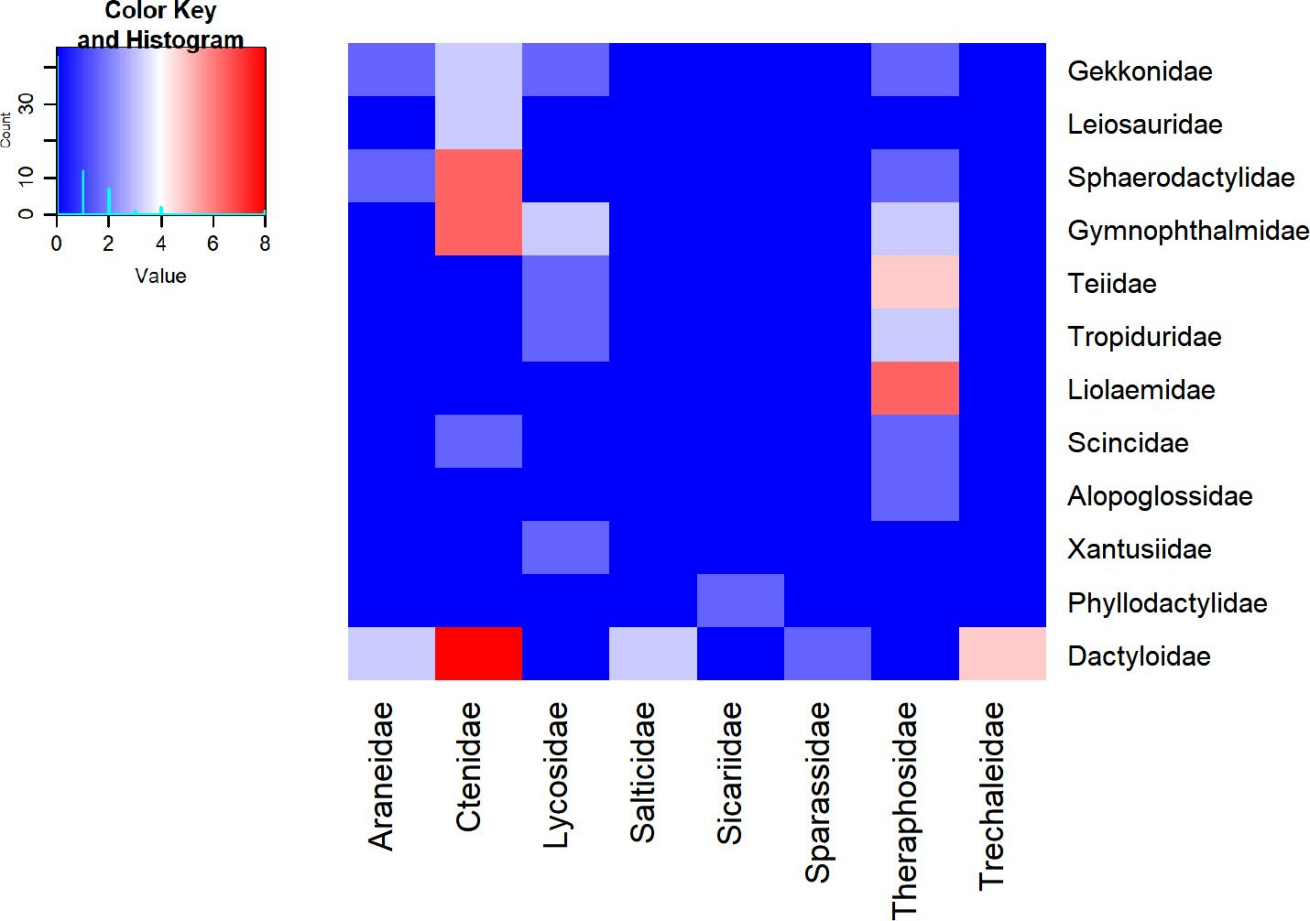
Heat map of predator–prey relationships between families of spiders (bottom) and lizards (right side). The small box shows the key color according to the observed number of predation reports

Predator–prey relationships between spider and lizard families are depicted in the heat map of Figure 3. The most salient corresponds to Ctenidae spiders consuming primarily Dactyloidae lizards, and Gymnophthalmidae and Sphaerodactylidae lizards as second in importance. Dactyloidae lizards corresponded exclusively to *Anolis* (Table 1), and this genus was also the most consumed by spiders of different families (representatives of five out of eight families; Figure 3). The second most frequent relationship between spider and lizard families corresponded to Theraphosidae spiders as the only consumers of Liolaemidae lizards. Theraphosids were the most generalist spiders, as they consumed the largest number of different lizard families (representatives of eight out of the twelve families; Figure 3).

**FIGURE 4:**
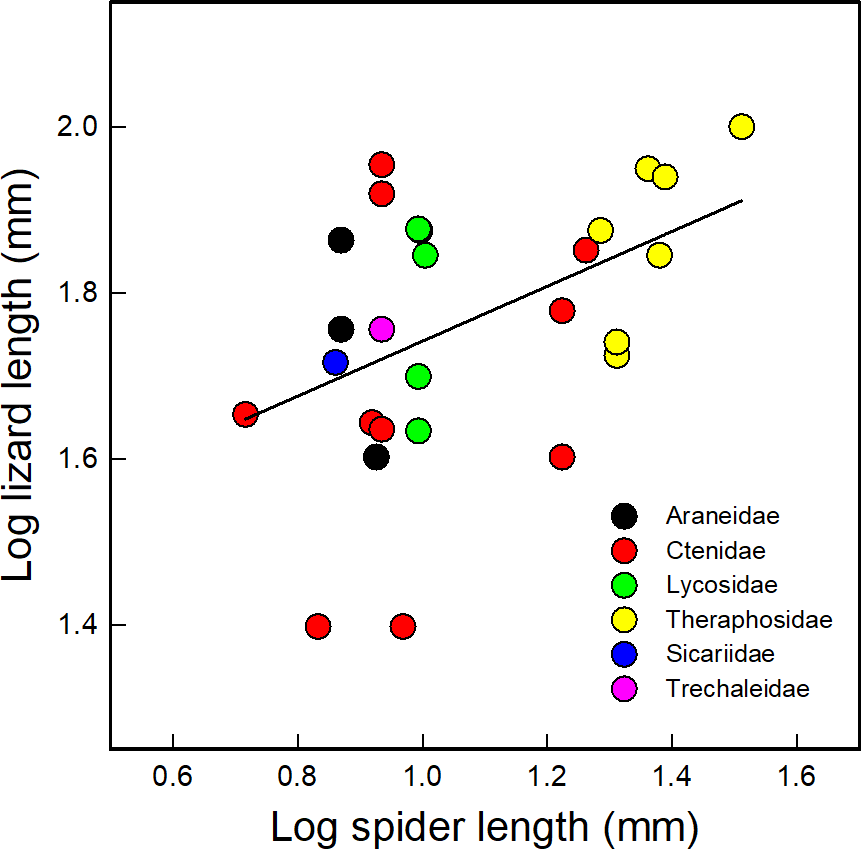
Correlation between body sizes of predators and prey (*N* = 28); carapace for spiders and snout–vent length for lizards. Data correspond to values reported in the literature (Table S1, see text) and not to the particular specimens reported in the studies listed in Table 1

We found a significant positive relationship between body sizes of predators and prey (*r* = .48, *p* = .017, *N* = 28; Figure 4), and spiders were significantly smaller than lizards (*t*_42_ = 14.08; *p *<< .001). There was, however, no association between the activity patterns (*p* = .51) and hunting strategies (*p* = .34) of spiders and lizards. The tendencies observed were a higher predation by nocturnal spiders upon diurnal lizards, and that the most predated lizards have ambush predator strategy, which were consumed mainly by ambush spiders, and secondly by active forager spiders (Table S1).

In addition to the predation events reported in the literature, we describe three new events, for which we did not collect or measure the animals.

**FIGURE 5:**
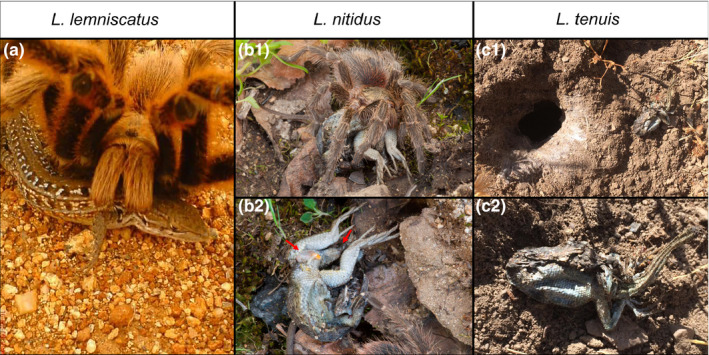
New cases of spider predation on *Liolaemus* lizards in Central Chile. (a) *Grammostola rosea* consuming an adult of *L. lemniscatus* at Cerro Mariposa. (b) Predation upon a juvenile of *L. nitidus* by a mygalomorph spider *Euathlus sp*.: bi) The spider holds the lizard with its chelicerae and pedipalps; bii) ventral view of the semidigested lizard body. The head, neck, and one frontal leg of the lizard were already consumed. In addition, the cloacal region and the tail show lacerations (red arrows). The observation occurred at Altos de Cantillana. (c) Partially digested individual of *L. tenuis* found close to a burrow of *G. rosea*, at Lo Barnechea: ci) Lizard corpse close to the spider burrow; cii) ventral view of the lizard. Photos: (a) Andrés Guajardo‐Santibáñez, (b) Bernardo Segura, and (c) Nicolás Zañartu

1. *Liolaemus lemniscatus* Gravenhorst, 1838: This observation was conducted in winter, 3 July 2009 (15:56 hr), at Cerro Mariposa, Central Chile (33°03ʹ59″S, 71°37ʹ60″W). We observed an adult of *L. lemniscatus* being caught by an adult of *Grammostola rosea* (Walckenaer, 1837) (Theraphosidae) (Figure 5a). The lizard was approaching the spider's burrow, while the resident was hidden inside, keeping its two frontal legs outside the burrow, moving them up and down. When the lizard was around 10 cm from the burrow, the spider came out and captured the lizard, sinking its fangs repetitively in its prey neck (Figure 5a). Subsequently, the spider dragged the prey inside the burrow, hiding it. The complete event lasted 90 s approximately.
2. *Liolaemus nitidus* (Wiegmann, 1834): This observation was conducted on spring, 21 September 2012 (14:50 hr), during an exploratory trip to Altos de Cantillana, Central Chile (33°54ʹ47″S, 71°02ʹ31″W). Underneath a stone, we found a juvenile of *L. nitidus* being eaten by a spider of the genus *Euathlus* Ausserer, 1875 (Theraphosidae) (Figure 5bi). The spider was holding the lizard with its fangs and pedipalps (Figure 5bi). Approximately after five minutes we found the spider, it released the lizard and moved a few centimeters away (Figure 5bii). The head, neck, and one of the forelimbs of the lizard showed signs of digestion, and its tail and cloaca were bruised (Figure 5bii).
3. *Liolaemus tenuis* (Duméril & Bibron 1837): The observation was made on summer, 20 February 2018 (_˜_11:30 hr), at Lo Barnechea, Central Chile (33°19ʹ07″S, 70°29ʹ13″W). We found the corpse of an adult of *L. tenuis* out of a burrow dwelt presumably by a tarantula *G. rosea* (Walckenaer 1837) (Figure 5ci). This assumption is based on the fact that in this area this is the only tarantula species that builds burrows with a circular shape on the ground and having an extension of web threads (Aguilera et al., 2019), as observed in Figure 5ci. The lizard was partially digested, and its tail was autotomized (Figure 5cii).

## 4 DISCUSSION

This is the first comprehensive review on spiders consuming lizards from the Neotropical and Andean regions. All reports were anecdotal observations and in most cases (89%), observations started when spiders were consuming their prey. Thus, for most cases it is unknown if spiders initiated the predation event, but scavenging has a limited occurrence in wild spiders, and this behavior has been only observed in laboratory settings, under induced conditions (Sandidge, 2003; Vickers et al., 2014; Wise, 1993). Therefore, we consider all the revised reports as true predation events.

Lizard predation by spiders has been mainly reported in the tropical areas of the Neotropical region, and few reports are located in the temperate areas of this region, or in the Andean region. This pattern can be accounted by three nonexclusive explanations: (1) The larger extension of the Neotropical relative to the Andean region provides bigger area to house more species, as predicted by the species–area relationship proposed by Connor and McCoy (2001). (2) The terrestrial biodiversity in the tropics is greater than in temperate environments, a latitudinal diversity gradient pattern that has been evaluated in numerous taxa (Hillebrand, 2004), and also in spiders and lizards from the Neotropical and Andean regions; species richness of these taxa decreases with latitude (Gainsbury & Meiri, 2017; Piel, 2018; Santos et al., 2017). (3) The preferential focus of ecological research on conservation hotspots or ecoregions in the Brazilian and Chacoan subregions (DeClerck et al., 2010; Mittermeier et al., 2005; Santos et al., 2011) provides more opportunities to observe interactions such as these of spiders consuming lizards. On the other hand, it is noteworthy that none of the available reviews on spiders consuming vertebrates (i.e., amphibians, bats, fish) include data from the Andean region (Menin et al., 2005; Nyffeler & Knörnschild, 2013; Nyffeler & Pusey, 2014; Valdez, 2020). As such, our review is the first in communicating vertebrate predation by spiders in this region.

Ctenidae spiders were the most predaceous species, particularly those of the genus *Ctenus*. Ctenids consumed mainly lizards of the families Dactyloidae and Gymnophthalmidae, which may result from their high diversity, abundance, and wide distribution in the Neotropical region (Midtgaard, 2020; Poe et al., 2017; Torres‐Carvajal et al., 2016). These spiders range from small to very large body sizes (Jocqué & Dippenaar‐Schoeman, 2006), and they are highly ubiquitous across the Neotropical rainforests (Gasnier et al., 1995; Pétillon et al., 2018). These nocturnal hunters ambush their prey on the foliage or on the soil surface (Jocqué & Dippenaar‐Schoeman, 2006; Schmitt et al., 1990), and may detect prey visually or by means of air or ground vibrations (Neuhofer et al., 2009). Although Ctenids’ diet is mainly composed by terrestrial invertebrates (Willemart & Lacava, 2017), there are numerous cases of these spiders consuming vertebrates such as fish (Nyffeler & Pusey, 2014), amphibians (Nyffeler & Altig, 2020; Salas et al., 2019; Valdez, 2020), and lizards (this review). In addition, it has been pointed out that because ctenids can reach high densities in the forest floor, vertebrate predation by these spiders can be ecologically important (Menin et al., 2005). Considering all these evidence, we postulate that ctenid spiders are common lizard predators in the Neotropics.

Theraphosids were the second more predaceous spider group after the ctenids, consuming the largest prey. In fact, theraphosids are the largest extant spiders (Foelix, 2011) and thus are important vertebrate predators (McCormick & Polis, 1982). These spiders have large chelicerae that allow them to capture prey of relative large size, such as snakes (Jorge et al., 2016), lizards (this review), birds (Brooks, 2012; Campos e Silva & de Meirelles, 2016), and mammals (Nyffeler & Knörnschild, 2013). In addition, theraphosids, as other spiders, inject toxic venom with their chelicerae that kill or paralyze relatively large prey (Foelix, 2011). On the other hand, theraphosids are the only spiders that consume Liolaemidae, as we report the first predation event by *G. rosea* and *Euathlus sp*. upon three *Liolaemus* species. The reported *Liolaemus* predators are vertebrates (i.e., snakes, lizards, raptors, and mammals; Jaksić et al., 1982; Jara & Pincheira‐Donoso, 2013; Reyes‐Olivares et al., 2017), and therefore, our reports are the first identifying invertebrates as *Liolaemus* predators. Moreover, *G. rosea* (Aguilera et al., 2019) and *Euathlus* spiders (Canals et al., 2012) are identified as consumers of terrestrial insects, and thus, our observations expand to vertebrates the range of prey consumed by these spiders.

Spiders have a generalist diet (Riechert & Harp, 1987), and data from the different reviews on spider predation upon vertebrates do not allow determining whether these predators do have any preferred vertebrate taxa as prey, and thus, the selective pressure that spiders may impose. The information of those studies is not comparable, that is, scientific literature versus scientific literature and data from the social media, in addition to cover dissimilar geographical scales and periods in their analyses (e.g., for anurans, see Menin et al., 2005; Nyffeler & Altig, 2020). Presently, however, based on Valdez (2020) it may be proposed that amphibians are a group highly consumed by spiders.

The activity patterns and hunting strategies of predators and prey did not show any significant association, which suggest that at least these two factors are not modulating the predator–prey interactions. Considering however, the trends that nocturnal spiders consumed diurnal lizards, and that ambush lizards were consumed by ambush predators, we propose that a larger sample size may confirm these trends.

The body sizes of spider predators and lizard prey showed a significant positive relationship, similarly to the one observed in other interactions with spiders as predators, and invertebrates (e.g., Bartos, 2011) and vertebrates, different from lizards (e.g., Menin et al., 2005), as prey; the bigger the predator, the bigger the prey. We also found that the spiders’ size was significantly smaller than that of the lizards, indicating that the hunting strategies used to capture lizards are efficiently enough to capture bigger prey. Although this relation should be taken cautiously because we did not use data from the actual individuals involved in the predatory events, these results suggest that spiders may ensure a higher energetic reward as compared to what can be obtained if a smaller prey is captured (Blamires et al., 2011).

The relevance of these spider‐lizard predator–prey interactions prompts for additional efforts to clarify the real impact that spider predation impose upon lizard populations (Nordberg et al., 2018), and thus, how strong the selective pressure of lizard consumption by spiders is. This, however, is a complex task, as, in fact, few studies have evaluated the effects that invertebrate predation may have on vertebrates (Nordberg et al., 2018; Toledo, 2005), due to the difficulty to observed these predation events. For instance, Nordberg et al. 2018), in more than 500 hr of field observations, only recorded five events of invertebrates consuming vertebrates. Moreover, the digestion mechanism of spiders (external oral digestion) renders impracticable prey identification by means of intestinal content analysis (Bauer, 1990; Cohen, 1998), unless stable isotope analysis or DNA extraction techniques from fecal samples are applied to identify the consumed prey (Akamatsu et al., 2004; Sint et al., 2015; Symondson, 2002). The use of these types of analyses, together with automated monitoring techniques as camera traps (Collett & Fisher, 2017), will allow evaluating the relevance of spiders consumption upon lizards in a noninvasive way, which nowadays is an important consideration due to the conservation problems that some spiders species are having (Leroy et al., 2013).

This review has allowed disclosing interaction patterns between spider predators and lizard prey in the Neotropical and Andean regions, where ctenids and theraphosids apparently may be the most relevant spider predators of lizards. Moreover, in some areas such as in the Chacoan subregion along the Atlantic coast, spiders may play a significant role in shape the lizards’ community structure, considering the high frequency of predation reports from this subregion. The identification of these invertebrates as lizard predators prompts for further quantitative studies on the importance of this type of trophic interactions in the communities of these regions. Quantify this impact will provide significant insights to understand the intraguild, and so bidirectional, interactions between spider and lizard populations, and how these modulate the different interactions at the community level. In addition, and highly relevant during the present Anthropocene, the understanding of these trophic relationships may also contribute in the design of conservation strategies for species that are threatened (Leroy et al., 2013; Van Winkel & Ji, 2012).

## CONFLICT OF INTEREST

The authors declare that they have no conflict of interest.

## AUTHOR CONTRIBUTIONS

**Claudio Reyes‐Olivares:** Conceptualization‐Lead, Data curation‐Lead, Formal analysis‐Lead, Investigation‐Lead, Methodology‐Lead, Project administration‐Lead, Resources‐Lead, Software‐Lead, Supervision‐Lead, Validation‐Lead, Visualization‐Lead, Writing‐original draft‐Lead, Writing‐review & editing‐Lead; **Andrés Guajardo‐Santibáñez:** Conceptualization‐Equal, Data curation‐Equal, Investigation‐Supporting, Validation‐Supporting, Writing‐review & editing‐Supporting; **Bernardo Segura:** Conceptualization‐Equal, Data curation‐Equal, Investigation‐Supporting, Validation‐Supporting, Writing‐review & editing‐Supporting; **Nicolás Zañartu:** Conceptualization‐Equal, Data curation‐Equal, Investigation‐Supporting, Validation‐Supporting, Writing‐review & editing‐Supporting; **Mario Penna:** Project administration‐Supporting, Supervision‐Supporting, Validation‐Supporting, Writing‐review & editing‐Supporting; **Antonieta Labra:** Conceptualization‐Lead, Data curation‐Lead, Formal analysis‐Lead, Funding acquisition‐Lead, Investigation‐Lead, Methodology‐Lead, Project administration‐Lead, Resources‐Lead, Software‐Lead, Supervision‐Supporting, Validation‐Lead, Visualization‐Lead, Writing‐original draft‐Lead, Writing‐review & editing‐Lead.

## Supporting information

Table S1

## Data Availability

All the data obtained in this review are presented in Table 1 and Supporting Information (Table S1).
