# Supplementary material for: Lizard predation by spiders: A review from the Neotropical and Andean regions
Source: Ecol Evol. 2020 Sep 22;10(20):10953–64. doi: 10.1002/ece3.6801 (PMC7593146; doi:10.1002/ece3.6801)
Supplement: Supplementary file 1 — Table S1 [file ECE3-10-10953-s001.pdf]

SUPPORTING INFORMATION

Lizard predation by spiders: a review from the Neotropical and Andean regions

Claudio Reyes-Olivares[1,2], Andrés Guajardo-Santibáñez[3], Bernardo Segura[4], Nicolás Zañartu[5], Mario Penna[2] and Antonieta Labra[6*]

1. Programa de Doctorado en Ciencias, con mención en Ecología y Biología Evolutiva, Facultad de Ciencias, Universidad de Chile, Santiago, Chile.

2. Laboratorio de Neuroetología, Instituto de Ciencias Biomédicas, Facultad de Medicina, Universidad de Chile, Santiago, Chile.

3. Calle René Lagos 403, Cerro Mariposa, Valparaíso, Chile.

4. Flora y Fauna Chile Ltda., Santiago, Chile.

5. Programa de Agronomía, Facultad de Agronomía e Ingeniería Forestal, Pontificia Universidad Católica de Chile, Santiago, Chile.

6. Centre for Ecological and Evolutionary Synthesis (CEES), Department of Biosciences, University of Oslo, Oslo, Norway.

*Correspondence:

Antonieta Labra. E-mail: a.l.lillo@bio.uio.no

Table S1. Data obtained from the literature of different characteristics of spider predators and lizard prey. Patterns of activity (diurnal vs. nocturnal) and foraging strategies (active vs. ambush predator) of predators and prey are listed. The body sizes of adult lizards (snout-vent length) and spiders (carapace length) are also reported. For predator and prey species in which sexual dimorphism in body size occurred, the size of both sexes (female/male) are reported. Superscripts indicate information sources.

**Lizard (Family/Species)**	**Lizard Habits/Foraging**[1]*	**Adult size (mm) ** (M/F)**	**Spider Family**	**Spider Species**	**Spider Habits/Foraging**	**Carapace length (mm) (M/F)**
**Alopoglossidae**						
Ptychoglossus bicolor	Diurnal[2]/Active	45[2]	**Theraphosidae**	*Pamphobeteus ferox*	Nocturnal/Ambush[3]	
**Dactyloidae**						
Anolis sp.	Diurnal/Ambush		**Salticidae**	*Phidippus sp.(bidentatus)*	Diurnal[4]/Ambush[5]r	5.6/5.9[6]

Anolis chrysolepis	Diurnal[7]/Ambush	75[8]	**Ctenidae**	*Ctenus sp.*	Nocturnal/Active[9]r	
Anolis fuscoauratus	Diurnal[10]/Ambush	50[11]	**Ctenidae**	*Ctenus sp.*	Nocturnal/Active[9]r	
Anolis gundlachi	Diurnal[12]/Ambush	68/45[13]	**Ctenidae**	*Oligoctenus ottleyi*	Nocturnal/Active[9]r	-/5.2[14]
Anolis humilis	Diurnal[10]/Ambush	44[11]	**Ctenidae**	*Kiekie curvipes*	Nocturnal/Active[9]r	8.7/8.3[15]
Anolis humilis	Diurnal[10]/Ambush	44[11]	**Trechaleidae**	*Cupiennius sp.*	Nocturnal/Ambush[16]r	
Anolis humilis	Diurnal[10]/Ambush	44[11]	**Ctenidae**	-	-	-
Anolis limifrons	Diurnal[10]/Ambush	48[11]	**Trechaleidae**	*Cupiennius sp.*	Nocturnal/Ambush[16]r	
Anolis limifrons	Diurnal[10]/Ambush	48[11]	**Salticidae**	-	-	-
Anolis porcatus	Diurnal[17]/Ambush	73[18]	**Araneidae**	*Argiope trifasciata*	Diurnal/Ambush[19]	3/7.4[20]
Anolis rodriguezii	Diurnal[87]/Ambush	40-45[87]	**Sparassidae**	-	-	-

Anolis sagrei	Diurnal[12]/Ambush	57[11]	**Araneidae**	*Argiope trifasciata*	Diurnal/Ambush[19]	3.0/7.4[20]
Anolis sagrei	Diurnal[12]/Ambush	57[11]	**Trechaleidae**	*Cupiennius cf. cubae*	Nocturnal/Ambush[16]r	7.5[21]/8.6[22]
**Gekkonidae**						
Hemidactylus mabouia	Nocturnal[23]/Ambush	75[11]	**Araneidae**	*Nephilengys cruentata*	Nocturnal/Ambush[24]r	2.0/9.9[24]
Hemidactylus mabouia	Nocturnal[23]/Ambush	75[11]	**Ctenidae**	-	-	-
Hemidactylus mabouia	Nocturnal[23]/Ambush	75[11]	**Lycosidae**	-	-	-
Hemidactylus mabouia	Nocturnal[23]/Ambush	75[11]	**Theraphosidae**	*Avicularia variegata*	Nocturnal/Ambush[25]r	19.4/19.4[26]
Hemidactylus frenatus	Nocturnal/Active[88]	60[88]	**Ctenidae**	*Phoneutria boliviensis*	Nocturnal/Ambush[89]	16.75[90]

**Gymnophthalmidae**						
Arthrosaura reticulata	Nocturnal[27]/Active	71[28]	**Ctenidae**	*Ancylometes rufus*	Nocturnal/Active[29]	18.8/18.3[30]
Cercosaura eigenmanni	Diurnal[31]/Active	50[32]	**Ctenidae**	*Ctenus sp.*	Nocturnal/Active[9]r	
Cercosaura schreibersii	Diurnal[33]/Active	50[32]	**Theraphosidae**	*Plesiopelma sp.*	Nocturnal/Ambush[34]r	
Cercosaura schreibersii	Diurnal[33]/Active	50[32]	**Lycosidae**	*Lycosa erythrognatha*	Nocturnal/Ambush[35]	9.37/9.85[36]
Ecpleopus gaudichaudii	Nocturnal[37]/Active	43.2[38]	**Ctenidae**	*Ctenus cf. ornatus*	Nocturnal/Active[9]r	9/8.6[39]
Loxopholis guianense	Diurnal[27]/Active	32.6[40]	**Ctenidae**	*Ctenus sp.*	Nocturnal/Active[9]r	
Micrablepharus atticolus	Diurnal[41]/Active	43[41]	**Lycosidae**	*Lycosa erythrognatha*	Nocturnal/Ambush[35]	9.37/9.85[36]

Micrablepharus maximiliani	Diurnal[42]/Active	39.8 [43]	**Theraphosidae**	-	-	-
**Leiosauridae**						
Enyalius bilineatus	Diurnal[44]/Ambush	90[44]	**Ctenidae**	*Ctenus ornatus*	Nocturnal/Ambush[45]r	9/8.6[39]
Enyalius perditus	Diurnal[46]/Ambush	83[46]	**Ctenidae**	*Ctenus ornatus*	Nocturnal/Ambush[45]r	9/8.6[39]
**Liolaemidae**						
Liolaemus lemniscatus	Diurnal[47]/Ambush	53[48]	**Theraphosidae**	*Grammostola rosea*	Nocturnal[49] /Active[50]	20.0/20.5[51]
Liolaemus nigroviridis	Diurnal[47]/Ambush	74[48]	**Theraphosidae**			
Liolaemus nitidus	Diurnal[47]/Ambush	88[48]	**Theraphosidae**	*Euathlus sp.*		
Liolaemus tenuis	Diurnal[47]/Ambush	55[48]	**Theraphosidae**	*Grammostola rosea*	Nocturnal[49] /Active[50]	20.0/20.5[51]

**Phyllodactylidae**						
Phyllodactylus gerrhopygus	Nocturnal[47]/Ambush-Active[91]	52[47]	**Sicariidae**	*Sicarius thomisoides*	Nocturnal/Ambush[92]	7.7/7.25[93]
**Scincidae**						
Plestiodon sumichrasti	Diurnal[52]/Active	96[11]	**Theraphosidae**	*Brachypelma sp.*	Nocturnal/Ambush[53]	
Scincella cherriei	Diurnal[54]/Active	63[55]	**Ctenidae**	*Anahita sp.*		
**Sphaerodactylidae**						
Chatogekko amazonicus	Diurnal[56]/Ambush	24[28]	**Ctenidae**	-	-	
Coleodactylus meridionalis	Diurnal[57]/Ambush	25[58]	**Ctenidae**	*Parabatinga brevipes*	Nocturnal/Active [59]r	7.1/6.8[60]
Coleodactylus	Diurnal[57]/Ambush	25[58]	**Ctenidae**	*Ctenus rectipes*	Nocturnal/Ambush[45]r	8.8/9.3[39]

meridionalis						
Gonatodes albogularis	Diurnal[61]/Ambush	40[11]	**Araneidae**	*Nephila clavipes*	Nocturnal/Ambush[62]	1.74/8.43[63]
Gonatodes albogularis	Diurnal[61]/Ambush	40[11]	**Ctenidae**	*Phoneutria boliviensis*	Nocturnal/Ambush[89]	16.75[90]
Lepidoblepharis xanthostigma	Nocturnal[64]/Ambush	38[11]	**Theraphosidae**	*Trichopelma sp.*		
**Teiidae**						
Aurivela longicauda	Diurnal[65]/Active	70[66]	**Lycosidae**	*Lycosa poliostoma*	Nocturnal/Active[67]	9.1/10.1[68]
Aurivela longicauda	Diurnal[65]/Active	70[66]	**Theraphosidae**	*Grammostola mendozae*	Nocturnal/Active[69]r	24/24[70]
Ameivula nigrigula	Diurnal[71]/Active	90[72]	**Theraphosidae**	*Lasiodora sp.*		
Kentropyx striata	Diurnal[73]/Active	100[73]	**Theraphosidae**	*Theraphosa cf. blondi*	Nocturnal/Active[74]	40/32.5[75]

**Tropiduridae**						
Tropidurus hispidus	Diurnal[76]/Ambush	124/89 [28]	**Theraphosidae**	*Lasiodora klugi*	Nocturnal/Ambush[77]r	23/23[78]
Tropidurus oreadicus	Diurnal[79]/Ambush	88.4/75.3[80]	**Lycosidae**	*Lycosa erytrognata*	Nocturnal/Ambush[35]	9.37/9.85[36]
Tropidurus semitaeniatus	Diurnal[81]/Ambush	87[82]	**Theraphosidae**	*Acanthoscurria natalensis*	Nocturnal[83]r/Ambush[84]	18.2/24.5[85]
**Xantusiidae**						
Lepidophyma tuxtlae	Nocturnal[86]/Ambush	97[11]	**Lycosidae**	*Hogna sp.*		

* Information on foraging mode was determined considering the foraging mode proposed for the family at which the genus belongs.

** = When sexual dimorphism was so marked, we provide the size for both sexes.

Foraging behavior: AC= active forager; AM= Ambush forager.

r = Data obtained for a species of the same genus because it was not possible to find information on the species

1. Reilly, McBrayer, & Miles, (2007); 2. Meza-Joya, Ramos-Pallares, & Hernández-Jaimes, (2014); 3. Amat-Garcia, Amat-Garcia, Andrade-C, & Rodriguez-Mahecha, (2007); 4. Rao & Díaz-Fleischer, (2012); 5. Richman & Jackson, (1992); 6. Edwards, (2004); 7. Vitt, Sartorius, Avila-Pires, & Espósito, (2001); 8. Kok, (1998); 9. Gasnier & Höfer, (2001); 10. Vitt, Avila-Pires, Zani, Sartorius, & Espósito, (2003); 11. Köhler, (2003); 12. Loew, Fleishman, Foster, & Provencio, (2002); 13. Schwartz & Henderson, (1991); 14. Petrunkevitch, (1930); 15. Polotow & Brescovit, (2018); 16. Schmitt, Schuster, & Barth, (1990); 17. Heselhaus & Schmidt, (1995); 18. Powell, (1992); 19. Blackledge, (1998); 20. Levi, (1968); 21. Barth & Cordes, (1998); 22. Fonseca-Hernández & Rodríguez-Cabrera, (2014); 23. Howard, Parmerlee, & Powell, (2001); 24. Kuntner, (2007); 25. Cloudsley-Thompson & Constantinou, (1985); 26. Fukushima & Bertani, (2017); 27. Hoogmoed & de Avila-Pires, (1989); 28. Vitt, Magnusson, Avila-Pires, & Lima, (2008); 29. Gasnier, Azevedo, Torres-Sanchez, & Höfer, (2002); 30. Höfer & Brescovit, (2000); 31. Vitt, Sartorius, Avila-Pires, & Espósito, (1998); 32. Carreira, Elena, & Meneghel Morena, (2005); 33. Entiauspe-Neto, Perleberg, & de Freitas, (2016); 34. Dias, Carvalho, Bonaldo, & Brescovit, (2009); 35. Moyano, (2008); 36. Torres de Arujo, (2006); 37. Eisemberg, Cassimiro, & Bertoluci, (2004); 38. Maia et al., (2011); 39. Brescovit & Simó, (2007); 40. Martins, (1991); 41. Rodrigues, (1996); 42. Rodrigues, (2003); 43. Dal Vechio, Recoder, Zaher, & Rodrigues, (2014); 44. Teixeira, Roldi, & Vrcibradic, (2005); 45. Höfer, Brescovit, & Gasnier, (1994); 46. Barreto-Lima, Pires, & Sousa, (2013); 47. Mella, (2017); 48. Mella, (2005); 49. Alfaro et al., (2013); 50. Canals, Salazar, Durán, Figueroa, & Veloso, (2007); 51. Pocock, (1899); 52. Campbell, (1998); 53. M’Rabet et al., (2007); 54. McCranie, Castaneda, & Nicholson, (2002);

55. Fitch, (1973); 56. Gamble, Daza, Colli, Vitt, & Bauer, (2011); 57. Ribeiro, Gogliath, Rodrigues, Barreto, & Freire, (2013); 58. Goncalves, Torquato, Skuk, & Sena, (2012); 59. Polotow & Brescovit, (2014); 60. Polotow & Brescovit, (2009); 61. Ellingson, Fleishman, & Loew, (1995); 62. Higgins & Buskirk, (1992); 63. Kuntner et al., (2018); 64. Groen, (2003); 65. Belver & Avila, (2001); 66. Scolaro, (2005); 67. Framenau et al., (2000); 68. Capocasale, (2001); 69. Bücherl, (1951); 70. Strand, (1907); 71. Arias, De Carvalho, Rodrigues, & Zaher, (2011); 72. Arias, de Carvalho, Zaher, & Rodrigues, (2014); 73. Mesquita, Costa, & Colli, (2006); 74. Carvalho, Norris, & Michalski, (2016); 75. de Pikelin & Schiapelli, (1966); 76. Vitt, Zani, & Caldwell, (1996); 77. Horta et al., (2005); 78. Mella-Laitao, (1922); 79. Meira, Faria, Silva, Miranda, & Zahn-Silva, (2007); 80. Rocha & Siqueira, (2008); 81. Ribeiro & Freire, (2010); 82. Ribeiro, Silva, & Freire, (2012); 83. Martins, Gallão, Bichuette, & Santos, (2016); 84. Pérez-Miles, Costa, Toscano-Gadea, & Mignone, (2005); 85. Lucas, Gonzalez Filho, Paula, Gabriel, & Brescovit, (2011); 86. Arenas-Moreno, Santos-Bibiano, Muñoz-Nolasco, Charruau, & Méndez-de la Cruz, (2018); 87. García-Balderas, Cedeño-Vázquez, & Mineros-Ramírez, (2016); 88. Hoskin, (2011); 89. Hazzi, (2014); 90. Pickard, (1897); 91. Pérez & Balta, (2011); 92. Taucare-Ríos, Veloso, Canals, & Bustamante, (2020); 93. Magalhaes, Brescovit, & Santos, (2017).

REFERENCES

Alfaro, C., Figueroa, D. P., Torres, H., Veloso, C., Venegas, F., Canals, L., & Canals, M. (2013). Effect of thermal acclimation on preferred temperatures in two mygalomorph spiders inhabiting contrasting habitats. *Physiological Entomology*, *38*, 20-25.

Amat-Garcia, G., Amat-Garcia, E., Andrade-C, M. G., & Rodriguez-Mahecha, J. V. (2007). *Libro rojo de los invertebrados terrestres de Colombia*. Conservación Internacional Colombia.

Arenas-Moreno, D. M., Santos-Bibiano, R., Muñoz-Nolasco, F. J., Charruau, P., & Méndez-de la Cruz, F. R. (2018). Thermal ecology and activity patterns of six species of tropical night lizards (Squamata: Xantusiidae: *Lepidophyma*) from Mexico. *Journal of Thermal Biology*, *75*, 97-105.

Arias, F., de Carvalho, C. M., Rodrigues, M. T., & Zaher, H. (2011). Two new species of *Cnemidophorus* (Squamata: Teiidae) of the *C. ocellifer* group, from Bahia, Brazil. *Zootaxa*, *3022*, 1-21.

Arias, F., de Carvalho, C. M., Zaher, H., & Rodrigues, M. T. (2014). A new species of *Ameivula* (Squamata, Teiidae) from southern Espinhaço mountain range, Brazil. *Copeia*, *2014*, 95-105.

Barreto-Lima, A. F., Pires, Ê. de O., & Sousa, B. M. (2013). Activity, foraging mode and microhabitat use of *Enyalius perditus* (Squamata) in a disturbed Atlantic rainforest in southeastern Brazil. *Salamandra*, *49*, 177-185.

Barth, F. G., & Cordes, D. (1998). *Cupiennius remedius* new species (Araneae, Ctenidae), and a key for the genus. *Journal of Arachnology*, *26*, 133-141.

Belver, L. C., & Avila, L. J. J. (2001). Ritmo de actividad diaria y estacional de *Cnemidophorus longicaudus* (Squamata: Teiidae: Teiinae) en el Norte de La Rioja, Argentina. *Boletin de la Sociedad de Biología de Concepción Chile, 72*, 31-36.

Blackledge, T. A. (1998). Stabilimentum variation and foraging success in *Argiope aurantia* and *Argiope trifasciata* (Araneae: Araneidae). *Journal of Zoology*, *246*, 21-27.

Brescovit, A. D., & Simó, M. (2007). On the Brazilian Atlantic Forest species of the spider genus *Ctenus* Walckenaer, with the description of a neotype for *C. dubius* Walckenaer (Araneae, Ctenidae, Cteninae). *Arachnology*, *14*, 1-18.

Bücherl, W. (1951). Estudos sobre o genero *Grammostola* Simon, 1892. *Monografías do Instituto Butantan, 1*, 1-203.

Campbell, J. A. (1998). *Amphibians and Reptiles of Northern Guatemala, the Yucatan, and Belize*. Oklahoma: University of Oklahoma Press.

Canals, M., Salazar, M. J., Durán, C., Figueroa, D., & Veloso, C. (2007). Respiratory refinements in the mygalomorph spider *Grammostola rosea* Walckenaer 1837 (Araneae, Theraphosidae). *The Journal of Arachnology*, *35*, 481-487.

Capocasale, R. M. (2001). Redescripción de *Lycosa poliostoma* (CL Koch)(Araneae, Lycosidae). *Revista Ibérica Aracnologia*, *3,* 79-86.

Carreira, S., Meneghel, M., & Achaval, F. (2005). *Reptiles de Uruguay*. Montevideo: Edición DIRAC.

Carvalho, W. D. de, Norris, D., & Michalski, F. (2016). Opportunistic predation of a Common Scale-backed Antbird (*Willisornis poecilinotus*) by a Goliath bird-eating spider (*Theraphosa blondi*) in the Eastern Brazilian Amazon. *Studies on Neotropical Fauna and Environment*, *51*, 239-241. https://doi.org/10.1080/01650521.2016.1237802

Cloudsley-Thompson, J. L., & Constantinou, C. (1985). Diurnal rhythm of activity in the arboreal tarantula *Avicularia avicularia* (L.) (Mygalomorphae: Theraphosidae). *Journal of Interdisciplinary Cycle Research*, *16*, 113-116. https://doi.org/10.1080/09291018509359879

Dal Vechio, F., Recoder, R., Zaher, H., & Rodrigues, M. T. (2014). Natural history of *Micrablepharus maximiliani* (Squamata: Gymnophthalmidae) in a Cerrado region of northeastern Brazil. *Zoologia (Curitiba)*, *31*, 114-118.

De Pikelin, G., & Schiapelli, R. (1966). Contribución al conocimiento de *Theraphosa leblondi* (Latreille), 1804 (Aranea: Theraphosidae). *Memórias do Instituto Butantan*, *33*, 667-674.

Dias, S. C., Carvalho, L. S., Bonaldo, A. B., & Brescovit, A. D. (2009). Refining the establishment of guilds in Neotropical spiders (Arachnida: Araneae). *Journal of Natural History*, *44*, 219-239. https://doi.org/10.1080/00222930903383503

Edwards, G. B. (2004). *Revision of the jumping spiders of the genus* Phidippus *(Araneae: Salticidae)* (Vol. 11). Florida Department of Agriculture and Consumer Services.

Eisemberg, C. C., Cassimiro, J., & Bertoluci, J. A. (2004). Notes on the diet the rare gymnophthalmid lizard *Ecpleopus gaudichaudii* from southeastern Brazil. *Herpetological Review*, *35*, 336-337.

Ellingson, J. M., Fleishman, L. J., & Loew, E. R. (1995). Visual pigments and spectral sensitivity of the diurnal gecko *Gonatodes albogularis*. *Journal of Comparative Physiology A*, *177*, 559-567.

Entiauspe-Neto, O., Perleberg, T., & de Freitas, M. A. (2016). Herpetofauna from an urban Pampa fragment in southern Brazil: Composition, structure and conservation. *Check List*, *12*, 1.

Fitch, H. S. (1973). A field study of Costa Rican lizards. *The University of Kansas Science Bulletin*, *50*, 39-126.

Fonseca-Hernández, E., & Rodríguez-Cabrera, T. M. (2014). Predation on a Cuban Brown Anole, *Anolis sagrei* (Dactyloidae), by a spider, *Cupiennius cubae* (Ctenidae), in the Cienfuegos botanical garden, South-central Cuba. *IRCF Journal Reptiles & Amphibians: Conservation and Natural History*, *21*, 98-99.

Framenau, V. W., Finley, L. A., Allan, K., Love, M., Shirley, D., & Elgar, M. A. (2000). Multiple feeding in wolf spiders: The effect of starvation on handling time, ingestion rate, and intercatch intervals in *Lycosa lapidosa* (Araneae: Lycosidae). *Australian Journal of Zoology*, *48*, 59-65.

Fukushima, C. S., & Bertani, R. (2017). Taxonomic revision and cladistic analysis of *Avicularia* Lamarck, 1818 (Araneae, Theraphosidae, Aviculariinae) with description of three new aviculariine genera. *ZooKeys*, (659), 1-185.

Gamble, T., Daza, J. D., Colli, G. R., Vitt, L. J., & Bauer, A. M. (2011). A new genus of miniaturized and pug-nosed gecko from South America (Sphaerodactylidae: Gekkota). *Zoological Journal of the Linnean Society*, *163*, 1244-1266.

García-Balderas, C. M., Cedeño-Vázquez, J. R., & Mineros-Ramírez, R. (2016). *Norops rodriguezii*. Predation. *Mesoamerican Herpetology*, *3*, 147-148.

Gasnier, T. R., & Höfer, H. (2001). Patterns of abundance of four species of wandering spiders (Ctenidae, *Ctenus*) in a forest in central Amazonia. *The Journal of Arachnology*, *29*, 95-103.

Gasnier, T. R., Azevedo, C. S. de, Torres-Sanchez, M. P., & Höfer, H. (2002). Adult size of eight hunting spider species in Central Amazonia: Temporal variations and sexual

dimorphisms. *The Journal of Arachnology, 30*, 146-154. https://doi.org/10.1636/0161-8202(2002)030[0146:ASOEHS]2.0.CO;2

Goncalves, U., Torquato, S., Skuk, G., & Sena, G. A. (2012). A new species of *Coleodactylus* Parker, 1926 (Squamata: Sphaerodactylidae) from the Atlantic Forest of northeast Brazil. *Zootaxa*, *3204*, 20-30.

Groen, M. (2003). *A comparative study of the herpetofauna in two different forest types at Caño Palma Biological Station, Costa Rica*. HAS Hogeschool.

Hazzi, N. A. (2014). Natural history of *Phoneutria boliviensis* (Araneae: Ctenidae): habitats, reproductive behavior, postembryonic development and prey-wrapping. *The Journal of Arachnology*, *42*, 303-310. https://doi.org/10.1636/Hi13-05.1

Heselhaus, R., & Schmidt, M. (1995). *Caribbean Anoles*. New Jersey: T.F.H. Publications, Inc.

Higgins, L. E., & Buskirk, R. E. (1992). A trap-building predator exhibits different tactics for different aspects of foraging behaviour. *Animal Behaviour*, *44*, 485-499.

Höfer, H., Brescovit, A. D., & Gasnier, T. (1994). The wandering spiders of the genus *Ctenus* (Ctenidae, Araneae) of Reserva Ducke, a rainforest reserve in central Amazonia. *Andrias, 13*, 81-98.

Höfer, Hubert, & Brescovit, A. D. (2000). A revision of the Neotropical spider genus *Ancylometes* Bertkau (Araneae: Pisauridae). *Insect Systematics & Evolution*, *31*, 323-360.

Hoogmoed, M. S., & de Avila-Pires, T. C. S. (1989). Observations on the nocturnal activity of lizards in a marshy area in Serra do Navio, Brazil. *Tropical Zoology, 2*, 165-173.

Horta, C. C. R., Chatzaki, M., Oliveira-Mendes, B. B. R., do Carmo, A. O., Siqueira, F. D. F., & Kalapothakis, E. (2005). The venom from *Lasiodora* sp.: A Mygalomorph

brazilianspider. In: P. Gopalakrishnakone, G. A. Corzo, M. E. de Lima, & E. Diego-García (Eds.). *Spider venom*. New York, NY: Springer-Verlag, New York Inc.

Hoskin, C. J. (2011). The invasion and potential impact of the Asian House Gecko (*Hemidactylus frenatus*) in Australia. *Austral Ecology*, *36*, 240-251. https://doi.org/10.1111/j.1442-9993.2010.02143.x

Howard, K. G., Parmerlee, J. S., & Powell, R. (2001). Natural history of the edificarian geckos *Hemidactylus mabouia*, *Thecadactylus rapicauda*, and *Sphaerodactylus sputator* on Anguilla. *Caribbean Journal of Science*, *37*, 285-287.

Köhler, G. (2003). *Reptiles of Central America*. Offenbach, Germany: Herpeton, Verlag Elke Köhler.

Kok, P. (1998). *Anolis nitens chrysopepsis* (goldenscale anole) predation. *Herpetological Review*, *29*, 41.

Kuntner, M. (2007). A monograph of *Nephilengys*, the pantropical 'hermit spiders' (Araneae, Nephilidae, Nephilinae). *Systematic Entomology*, *32*, 95-135.

Kuntner, M., Hamilton, C. A., Cheng, R.-C., Gregorič, M., Lupše, N., Lokovšek, T., … Bond, J. E. (2018). Golden orbweavers ignore biological rules: Phylogenomic and comparative analyses unravel a complex evolution of sexual size dimorphism. *Systematic Biology, 68*, 555-572. https://doi.org/10.1093/sysbio/syy082

Levi, H. W. (1968). The spider genera *Gea* and *Argiope* in America (Araneae: Araneidae). *Bulletin of the Museum of Comparative Zoology*, *136*, 319-352.

Loew, E. R., Fleishman, L. J., Foster, R. G., & Provencio, I. (2002). Visual pigments and oil droplets in diurnal lizards. *Journal of Experimental Biology*, *205*, 927-938.

Lucas, S. M., Gonzalez Filho, H. M., Paula, F. dos S., Gabriel, R., & Brescovit, A. D. (2011). Redescription and new distribution records of *Acanthoscurria natalensis* (Araneae: Mygalomorphae: Theraphosidae). *Zoologia (Curitiba)*, *28*, 525-530.

Maia, T., Almeida-Gomes, M., Siqueira, C. C., Vrcibradic, D., Kiefer, M. C., & Rocha, C. F. (2011). Diet of the lizard *Ecpleopus gaudichaudii* (Gymnophthalmidae) in Atlantic Rainforest, state of Rio de Janeiro, Brazil. *Zoologia*, *28*, 587-592 . https://doi.org/10.1590/S1984-46702011000500006.

Magalhaes, I. L., Brescovit, A. D., & Santos, A. J. (2017). Phylogeny of Sicariidae spiders (Araneae: Haplogynae), with a monograph on Neotropical *Sicarius*. *Zoological Journal of the Linnean Society*, *179*, 767-864. https://doi.org/10.1111/zoj.12442

Martins, A. L., Gallão, J. E., Bichuette, M. E., & Santos, E. F. (2016). The first record of *Notocyphus tyrannicus* Smith,(hymenoptera: Pompilidae) as parasitoid of *Acanthoscurria Ausserer*, 1871 (Teraphosidae: Teraphosinae). *Brazilian Journal of Biology*, *76*, 806-807.

Martins, M. (1991). The lizards of Balbina, Central Amazonia, Brazil: A qualitative analysis of resource utilization. *Studies on Neotropical Fauna and Environment*, *26*, 179-190.

McCranie, J. R., Castaneda, F. E., & Nicholson, K. E. (2002). Preliminary results of herpetofaunal survey work in the Rus Rus region, Honduras: A proposed biological reserve. *Herpetological Bulletin*, *81*, 22-29.

Meira, K. T. R., Faria, R. G., Silva, M. das D. M., Miranda, V. T. de, & Zahn-Silva, W. (2007). História natural de *Tropidurus oreadicus* em uma área de cerrado rupestre do Brasil Central. *Biota Neotropica*, *7*, 155-163.

Mella, J. (2005). *Guía de campo de reptiles de Chile: Zona central* (A. P. G. Peñaloza, F. Novoa, & M. Contreras, Eds.). Santiago, Chile: Ediciones del Centro de Ecología Aplicada Ltda.

Mella, J. (2017). *Guía de campo de reptiles de Chile. Tomo 1: Zona central* (A. P. G. Peñaloza, Ed.). Santiago.

Mella-Laitao. (1922). Theraphosoideas do Brasil. *Revista do Museu Paulinista*, 13, 1-438.

Mesquita, D. O., Costa, G. C., & Colli, G. R. (2006). Ecology of an Amazonian savanna lizard assemblage in Monte Alegre, Pará state, Brazil. *South American Journal of Herpetology*, *1*, 61-71. https://doi.org/10.2994/1808-9798(2006)1[61:EOAASL]2.0.CO;2

Meza-Joya, F. L., Ramos-Pallares, E., & Hernández-Jaimes, C. (2014). *Ptychoglossus bicolor*. *Catálogo de Anfibios y Reptiles de Colombia*, *2*, 24-29.

Moyano, R. D. (2008). *Aracnoidismo: Arañas y escorpiones de importancia médica en Argentina*. Buenos Aires: Literature of Latin America.

M'Rabet, S. M., Hénaut, Y., Sepúlveda, A., Rojo, R., Calmé, S., & Geissen, V. (2007). Soil preference and burrow structure of an endangered tarantula, *Brachypelma vagans* (Mygalomorphae: Theraphosidae). *Journal of Natural History*, *41*, 1025-1033. https://doi.org/10.1080/00222930701384547

Pérez, J., & Balta, K. (2011). Ecología de *Phyllodactylus angustidigitus* y *P. gerrhopygus* (Squamata: Phyllodactylidae) de la Reserva Nacional de Paracas, Perú. *Revista peruana de Biología*, *18*, 217-223.

Pérez-Miles, F., Costa, F. G., Toscano-Gadea, C., & Mignone, A. (2005). Ecology and behaviour of the 'road tarantulas' *Eupalaestrus weijenberghi* and *Acanthoscurria suina* (Araneae, Theraphosidae) from Uruguay. *Journal of Natural History, 39,* 483-498.

Petrunkevitch, A. (1930). *The spiders of Porto Rico: Sub-order Arachnomorphae*. Connecticut Academy of Arts and Sciences.

Pickard, F. O. (1897) VII.-On cteniform spiders from the Lower Amazons and other regions of North and South America, with list of all known species of these groups hitherto recorded from the New World. *Annals and Magazine of Natural History*, *19*, 52-106. http://dx.doi.org/10.1080/00222939708680507

Pocock, R. I. (1899). A new stridulating theraphosid spider from South America. *Annals and Magazine of Natural History*, *3*, 347-349.

Polotow, D., & Brescovit, A. D. (2009). Revision and cladistic analysis of *Isoctenus* and description of a new neotropical genus (Araneae, Ctenidae, Cteninae). *Zoological Journal of the Linnean Society*, *155*, 583-614.

Polotow, D., & Brescovit, A. D. (2014). Phylogenetic analysis of the tropical wolf spider subfamily Cteninae (Arachnida, Araneae, Ctenidae). *Zoological Journal of the Linnean Society*, *170*, 333-361.

Polotow, D., & Brescovit, A. D. (2018). *Kiekie,* a new Neotropical spider genus of Ctenidae (Cteninae, Araneae). *Zootaxa*, *4531*, 353-373.

Powell, R. (1992). *Anolis porcatus*. *Catalogue of American Amphibians and Reptiles (CAAR),* 541.1.

Rao, D., & Díaz-Fleischer, F. (2012). Characterisation of predator-directed displays in Tephritid flies. *Ethology*, *118*, 1165-1172. https://doi.org/10.1111/eth.12021

Reilly, S. M., McBrayer, L. B., & Miles, D. B. (2007). *Lizard ecology: The evolutionary consequences of foraging mode*. New York.: Cambridge University Press.

Ribeiro, L. B., Gogliath, M., Rodrigues, R. G., Barreto, R. M. F., & Freire, E. M. X. (2013). Two new records of *Coleodactylus meridionalis* (Boulenger, 1888) (Squamata,

Sphaerodactylidae) in north-eastern Brazil, including a map and comments regarding its geographical distribution. *Herpetology Notes*, *6*, 23-27.

Ribeiro, L. B., & Freire, E. M. (2010). Thermal ecology and thermoregulatory behaviour of *Tropidurus hispidus* and *T. semitaeniatus* in a caatinga area of northeastern Brazil. *The Herpetological Journal*, *20,* 201-208.

Ribeiro, L. B., Silva, N. B., & Freire, E. M. (2012). Reproductive and fat body cycles of *Tropidurus hispidus* and *Tropidurus semitaeniatus* (Squamata, Tropiduridae) in a caatinga area of northeastern Brazil. *Revista Chilena de Historia Natural, 85*, 307-320.

Richman, D. B., & Jackson, R. R. (1992). A review of the ethology of jumping spiders (Araneae, Salticidae). *Bulletin of the British arachnological Society, 9*, 33-37.

Rocha, C. F. D., & Siqueira, C. C. (2008). Feeding ecology of the lizard *Tropidurus oreadicus* Rodrigues 1987 (Tropiduridae) at Serra dos Carajás, Pará state, northern Brazil. *Brazilian Journal of Biology*, *68,* 109-113.

Rodrigues, M. T. (1996). A new species of lizard, genus *Micrablepharus* (Squamata: Gymnophthalmidae), from Brazil. *Herpetological Review*, *52*, 535-541.

Rodrigues, M. T. (2003). Herpetofauna da caatinga. In: I. R. Leal, M. Tabarelli, & J. M. Cardoso da Silva (Eds.). *Ecologia e conservação da Caatinga* (Vol. 1, pp. 181-236). Recife: Universidade Federal de Pernambuco.

Schmitt, A., Schuster, M., & Barth, F. G. (1990). Daily locomotor activity patterns in three species of *Cupiennius* (Araneae, Ctenidae): The males are the wandering spiders. *The Journal of Arachnology*, *18,* 249-255.

Schwartz, A., & Henderson, R. W. (1991). *Amphibians and reptiles of the West Indies: Descriptions, distributions, and natural history*. Gainesville, FL, USA: University Press of Florida.

Scolaro, A. (2005). *Reptiles patagónicos sur: Una guía de campo*. Trelew: Universidad Nacional de la Patagonia San Juan Bosco.

Strand, E. (1907). Aviculariidae und Atypidae des Kgl. Naturalienkabinetts in Stuttgart. *Jahreshefte des Vereins für vaterländische Naturkunde in Württemberg*, *63*, 1-100.

Taucare-Ríos, A., Veloso, C., Canals, M., & Bustamante, R. O. (2020). Daily thermal preference variation of the sand recluse spider *Sicarius thomisoides* (Araneae: Sicariidae). *Journal of Thermal Biology*, *87*, 102465. https://doi.org/10.1016/j.jtherbio.2019.102465

Teixeira, R. L., Roldi, K., & Vrcibradic, D. (2005). Ecological comparisons between the sympatric lizards *Enyalius bilineatus* and *Enyalius brasiliensis* (Iguanidae, Leiosaurinae) from an Atlantic Rain-Forest area in southeastern Brazil. *Journal of Herpetology*, *39*, 504-510.

Torres de Arujo, R. R. (2006). *Tamanho do corpo em aranhas Lycosidae e Ctenidae (Arachnida: Aranae): O papel do dimorfismo sexual e das variações sazonais*. Brasilia, Brazil: Universidad de Brasilia.

Vitt, L. J., Magnusson, W. E., Avila-Pires, T. C. S., & Lima, A. P. (2008). *Guide to the lizards of Reserva Adolpho Ducke, Central Amazonia*. São Paulo: Áttema.

Vitt, L. J., Avila-Pires, T. C. S., Zani, P. A., Sartorius, S. S., & Espósito, M. C. (2003). Life above ground: Ecology of *Anolis fuscoauratus* in the Amazon rain forest, and comparisons with its nearest relatives. *Canadian Journal of Zoology*, *81*, 142-156.

Vitt, L. J., Sartorius, S. S., Avila-Pires, T. C. S., & Espósito, M. C. (1998). Use of time, space, and food by the gymnophthalmid lizard *Prionodactylus eigenmanni* from the western Amazon of Brazil. *Canadian Journal of Zoology*, *76*, 1681-1688. https://doi.org/10.1139/z98-115

Vitt, L. J., Sartorius, S. S., Avila-Pires, T. C. S., & Espósito, M. C. (2001). Life on the leaf litter: The ecology of *Anolis nitens tandai* in the Brazilian Amazon. *Copeia*, *2001*, 401-412. https://doi.org/10.1643/0045-8511(2001)001[0401:LOTLLT]2.0.CO;2

Vitt, L. J., Zani, P. A., & Caldwell, J. P. (1996). Behavioural ecology of *Tropidurus hispidus* on isolated rock outcrops in Amazonia. *Journal of Tropical Ecology*, *12*, 81-101.
